# Corrosion of Chromium Coating Fabricated on Zircaloy-4 Substrate

**DOI:** 10.3390/ma17184445

**Published:** 2024-09-10

**Authors:** Florentina Golgovici, Diana Diniași, Paul Pavel Dincă, Bogdan Butoi, Ioana Demetrescu

**Affiliations:** 1 Department of General Chemistry, National University of Science and Technology Politehnica Bucharest, Splaiul Independentei Street, No. 313, 060042 Bucharest, Romania; florentina.golgovici@upb.ro (F.G.);; 2 Institute for Nuclear Research Pitesti, Campului Street, No. 1, 115400 Mioveni, Romania; 3 National Institute for Laser, Plasma and Radiation Physics, Atomistilor Street, No. 409, 077125 Magurele, Romania; paul.dinca@inflpr.ro (P.P.D.); bogdan.butoi@inflpr.ro (B.B.); 4 Academy of Romanian Scientists, 3 Ilfov, 050094 Bucharest, Romania

**Keywords:** corrosion, chromium coating, magnetron sputtering, accident-tolerant materials

## Abstract

In the nuclear industry, coated cladding is a topical problem and it is chosen as the near-term and most promising ATF (Accident-Tolerant Fuel) cladding concept. The main objective of this concept is to enhance the accident tolerance of nuclear power plants and accordingly, the performance of cladding is expected to be improved. This work assesses the corrosion performance of a Zircalloy-4 alloy coated with a thin chromium coating by MS (magnetron sputtering), tested under a CANDU (CANada Deuterium Uranium) reactor primary circuit simulated condition (LiOH solution, 10 MPa, 310 °C, pH = 10.5). The anticorrosive performance is evaluated by a gravimetric analysis, a metallographic analysis, X-ray diffraction, electronic microscopy, and electrochemical methods. A four times less gain mass was noticed compared to uncoated Zircaloy-4, indicating a smaller corrosion rate. The SEM micrographs illustrate that the coatings are still adherent, and they are keeping the initial morphological characteristics during the autoclaving process. A SEM cross-section analysis shows values of the thickness of the coatings between 0.8 and 1.46 µm. By XRD, the presence of Cr_2_O_3_ oxide is identified. Electrochemical testing confirms good stability and good corrosion performance of Cr coating over time under autoclave conditions.

## 1. Introduction

Zircaloy-4 is a zirconium alloy with remarkable characteristics such as corrosion resistance, mechanical strength, heat transfer, low neutron-capture cross-section, and optoelectronic properties. For such properties, zirconium-based materials are well known in multiple industrial applications such as electronics, biomedical, and energy, primarily in nuclear power plants where they are used as cladding material for fuel rods. The microelectronic industry has employed notable use of zirconium due to its high dielectric constant, stability in aggressive harsh environments, and employment as a valve metal [1].

Having biocompatibility and a low ion release in bioenvironments, Zr and its alloys were tested successfully as biomaterial [2]. Due to its native passive oxide, ZrO_2_ is an effective protective layer, with increased action after anodization [3]. After the 2011 disaster at the Fukushima Daiichi plant, which showed low resistance of nuclear materials such as fuel element claddings, international research efforts were focused on the development of other safety materials as well as a new concept of protective coatings developed on existing cladding element materials [4]. Such a trend led to the choice of the type of coating and its structure, and properties to meet the requirements for the ATF materials. Corrosion on the outer side of the fuel rod and hydrogen absorption can cause a drastic decrease in their performance under the CANDU (CANada Deuterium Uranium) nuclear power plant condition [5]. Therefore, a near-term solution for increasing the performance of already existing Zr alloys (Zircalloy) is the development of various types of coatings deposited by multiple deposition techniques [6], resulting in ATF (Accident-Tolerant Fuel) materials. The best results were obtained for metallic Cr coatings developed by physical deposition techniques [7]. However, the increasing cladding performance, both under normal operation and simulated accident conditions, depends on the properties of the coatings [8].

Metallic Cr coatings are considered the most promising ATF coatings for fuel cladding [9,10,11,12]. Due to the development of chromium oxide, these materials have a high melting point, a thermal expansion coefficient like zirconium alloys, and an excellent resistance to corrosion in water and steam [13]. These are the major characteristics that they meet. Such coatings have been produced using several kinds of techniques, including electroplating, magnetron sputtering, cold spraying, cathodic arc deposition, and 3D laser cladding. The involvement of research/industrial centers like CEA (France), VNIINM (Russia), KIT (Germany), and others attests to the huge potential of these coatings.

Until now, some comprehensive examinations have also been carried out in nuclear reactors with claddings coated with metallic chromium coatings and UO_2_ fuel. The correlation between the structure and properties of coatings depends on the deposition method and can be monitored by process parameters for better performance.

Physical vapor deposition (PVD) methods are chosen due to a relative low deposition temperature and low oxidation rate of the substrate [14]. However, it is desired to obtain homogeneous structures of the coating, instead of the columnar ones, specific to these methods [15]. Thus, several methods are identified to improve the microstructural and mechanical properties of the coating, such as the optimization of the deposition methods and working parameters, respectively, and the application of pre-treatments to the substrate and the coating [16]. Also, studies on the evaluation of the potential of the coatings to produce impurities and more in-pile and representative reactor condition tests are required.

As a critical analysis of the state of the art of Cr coatings on a Zircaloy-4 substrate, it is important to mention that the existing literature research was not oriented to corrosion in simulated conditions in a working reactor. In this regard, this paper will provide new experiments on current perspectives and discussions on advanced coating deposited on a Zircaloy-4 substrate by magnetron sputtering, as well as its corrosion mechanisms under static conditions for the long term. Using a large kind of characterization test for this Cr coating, the present manuscript denotes the original character and could be an indicator for the future selection of improving materials for nuclear energy.

## 2. Materials and Methods

### 2.1. Materials

Chromium coatings were applied to Zircaloy-4 alloy samples using the continuous current magnetron sputtering deposition (DC-MS) technique. The coating deposition procedure was carried out in a 60 cm diameter circular chamber. To reduce residual gas contamination in the resultant coatings, a turbomolecular pump (≤10^−9^ bar ± 10%) was used to provide ultrahigh vacuum conditions before deposition. In advance of the start of the deposition procedure, the substrates were cleaned using an ultrasonic technique involving a chemical bath made up of an acetone and isopropyl alcohol solution. The purpose of this step was to remove any macroscopic impurities as well as mechanical processing debris. The cleaning process lasted 15 min at a frequency of 45 kHz, followed by thorough washing with distilled water and drying. The cleaned substrates were then placed at a predetermined distance of 10 cm in front of the two sputtering sources on a solid support.

The used device consisted of two water-cooled magnetron heads equipped with high-purity-grade chromium targets (99.95%) of a 2 mm thickness and 50 mm diameter. Both cathodes were operated in the DC mode. One of the cathodes was positioned at a 45° incidence to the Zircaloy-4 substrates while the other was positioned at a normal incidence; at the same time, the solid holder where the samples were placed was rotated, ensuring a good coating uniformity despite the curved shape of the substrates. Once a base pressure of 10^−9^ bar was reached, Argon (Ar) gas of higher purity was introduced into the reaction chamber at a flow rate of 20 mL/min ± 2% through a calibrated mass flow controller, raising the pressure of 10^−5^ bar. By biasing the substrates (−200 V), a glow discharge was created using the Ar gas to remove surface oxides and impurities that attached to the substrates after the cleaning process (dust). To clean the Cr targets, remove impurities, and guarantee a strong link between the deposited layer and the substrate, an obturator was finally positioned between the sputtering source and the deposition substrates. This step was essential considering the low roughness of the Zircaloy-4 substrates.

### 2.2. Autoclave Testing

Samples of Zy-4/Cr_MS (Zircaloy-4 alloy deposited with Cr by MS) were washed, degreased, dried, and weighed for oxidation testing on both sides. The autoclave testing was carried out in a 1 L static autoclave, under simulated standard CANDU primary circuit conditions (LiOH, pH = 10.5, 360 °C, and 100 atm). Every 504 h, three samples were weighed, and some of them were retained to perform a further analysis.

### 2.3. Methods for Corrosion Assessment

The samples were characterized before and after the autoclave test. After testing, some of the samples were cross-sectioned into smaller parts and embedded in cupric resin for further characterization. To evaluate the anticorrosive performance of the tested samples, the following tests were applied: a gravimetric analysis, a metallographic analysis, X-ray diffraction, SEM, EDS, open-circuit potential variation, potentiodynamic polarization, and electrochemical impedance spectroscopy.

The metallographic analysis was performed using the Olympus BX51M optical microscope (Olympus Corporation, Tokyo, Japan) to highlight Vickers microhardness. SEM/EDS investigations were carried out using a HITACHI SU5000 field emission scanning electron microscope (Hitachi, Tokyo, Japan) equipped with an energy-dispersive X-ray analyzer (EDS, Oxford Instruments, Oxford, UK) operated at an accelerating voltage of 30 kV.

XRD patterns were obtained using a SmartLab X Ray Diffractometer (Rigaku Corporation, Tokyo, Japan) using CuKα radiation (*λ* = 1.5406 Å) and operating at room temperature. X-ray diffraction measurements of all the samples were carried out in the 20–100° range. The identification of the phase was made by referring to the International Center for Diffraction Data—ICDD (PDF-2) database.

Electrochemical measurements were performed using a PARSTAT 2273 computer-controlled electrochemical measurement system (Princeton Applied Research, AMETEK, Oak Ridge, TN, USA) with a conventional three-electrode electrochemical cell consisting of a working electrode (Zy-4 sample), a saturated calomel reference electrode (SCE), and two auxiliary electrodes (graphite rods). The electrochemical tests were carried out at room temperature (22 ± 2 °C). Electrochemical impedance spectroscopy measurements were performed in a chemically inert solution with pH = 7.26 (0.05 M boric acid with 0.001 M borax solution), which did not affect the oxide layer features. For electrochemical impedance spectroscopy tests and open-circuit potential measurements, a LiOH solution with a pH of 10.5 was used as the electrolyte.

Potentiodynamic measurements were made at room temperature at a scan rate of 0.5 mV·s^−1^ and a range from −250 to 1000 mV relative to the open-current potential (OCP), in a specific primary circuit solution (LiOH solution, pH 10.5). It is remarked that the “scan rate” had a significant effect in the attained distortions in the potentiodynamic polarization curves, as previously reported in study [17]. The behavior of the material with generalized corrosion was observed by open-circuit potential measurements in the same electrolyte.

Electrochemical impedance spectroscopy (EIS) tests were performed at open-circuit potential (OCP) with an amplitude of 10 mV in the frequency range from 100 mHz to 100 kHz after OCP stabilization. To obtain quantitative data, the experimental EIS results were simulated with equivalent electrical circuits as appropriate models using ZView 2.90c software (Scribner Associates Inc., Southern Pines, NC, USA).

Electrochemical impedance investigations (EISs) are based on Nyquist, and Bode diagrams. The experimental EIS data are fitted with the well-known equivalent circuit model (EC) [18,19,20].

Each electrochemical corrosion test was repeated at least three times.

Two types of solution have been used for the electrochemical tests, as follows: a LiOH solution with pH = 10.5 (similar to a primary circuit solution of a CANDU nuclear reactor) and a chemically inert solution (to not disturb the layer properties) with pH = 7.15 (0.05 M boric acid with 0.001 M borax solution).

**Figure 1:**
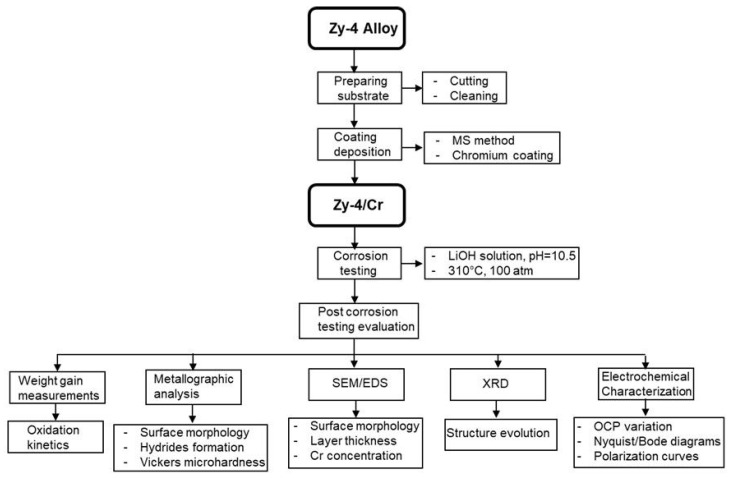
Research diagram.

Figure 1 presents a research diagram that details the chosen algorithm and study phases.

## 3. Results

### 3.1. Gravimetric Analysis of Cr-Coated Zircaloy-4 Alloy, Examined under Primary Circuit Situations

Weight gain measurements of Cr-coated Zircaloy-4 alloy samples indicate smaller corrosion rates than the uncoated Zircaloy-4 alloy [21]. Every 504 h up to 3024 h, gravimetric measurements on representative samples (without exfoliation/delamination) are illustrated in Figure 1.

**Figure 2:**
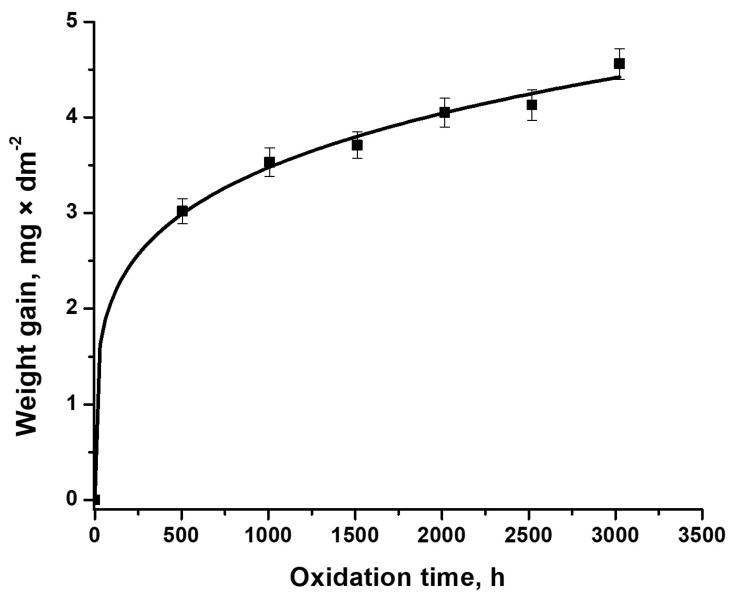
Corrosion weight gain versus oxidation time for Cr-coated Zircaloy-4 alloy tested under primary circuit conditions.

Figure 2 presents a mass gain of about 4.56 mg/dm^2^ after 3024 h of autoclave testing, about four times less compared to the uncoated Zircaloy-4 samples [21]. It is also observed that there is a fairly stable corrosion behavior, the difference in mass gain between the end of the autoclave testing process and the first 504 h, being only about 1.5 mg/dm^2^.

It is known that a transition in the corrosion kinetics of zirconium alloys takes place [11]. This transition occurs when the oxide film reaches around 2–3 µm [11]. After this, the corrosion rate of zirconium alloys increases sharply, following linear kinetics, called post-transition corrosion [22,23]. In this study, the corrosion kinetics was placed in the pre-transition stage.

For corrosion process characterization, the weight gain curve was fitted according to the following equation [24]:

(1) $$\Delta W=k_{p}*t^{n}$$

where ΔW is the oxide weight gain (mg/dm^2^), *k_p_* is the rate constant, *t* is the exposure time (h), and *n* is the exponent. The last two factors are dependent on the alloy. The parameters’ kinetics obtained is displayed in Table 1.

The power equation has been fitted very well with the gain weight measurements, obtaining a confidence factor of R^2^ = 0.997. The values of both A and n constants are smaller compared to the values obtained for the uncoated Zircaloy-4 alloy [21], which indicates lower corrosion kinetics for the coated Zircaloy-4 alloy. As shown in Figure 2, the evolution of the weight gain mass after the first and last autoclave cycle suggests a slight acceleration of the corrosion kinetics, compared to the intermediate period. It can be hypothesized that after the first 504 h of autoclave testing, a thin Cr_2_O_3_ layer was formed, which acts as a diffusion barrier layer. The slightly increased shift in mass gain after 3024 h of autoclave testing may suggest a decrease in the anticorrosive properties of the Cr coating.

### 3.2. Morphological and Structural Characterization of Cr Coating Post-Autoclave Testing

#### 3.2.1. Metallographic Analysis

The hydrogen resulting from the corrosion reaction on the outer part of the cladding is absorbed in the cladding material. The fraction of absorbed hydrogen and its solubility grow as temperature and exposure time increase [25,26]. It was noticed that there was an excess of hydrogen precipitates, leading to the formation of zirconium hydrides (ZrH_x_), which decrease the mechanical properties of the material, inducing embrittlement and cracking it.

**Figure 3:**
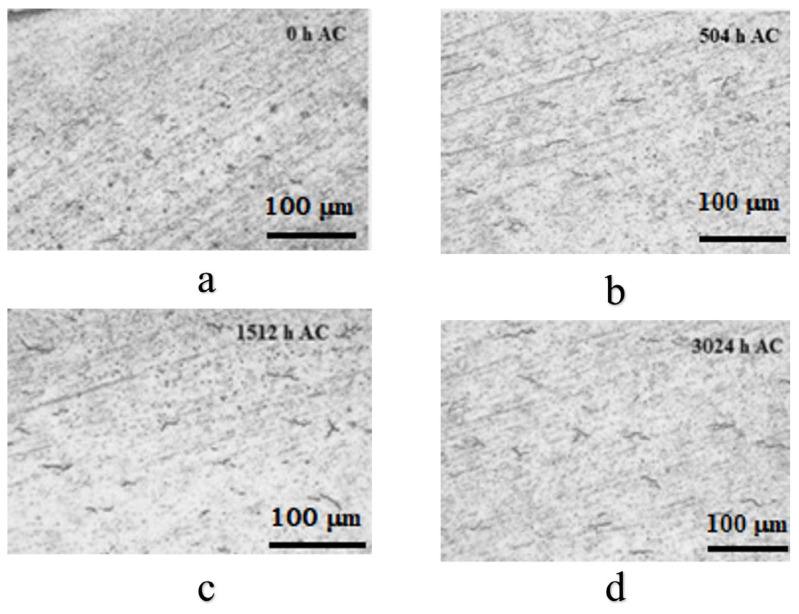
Representative zirconium hydride formation for Cr-coated Zircaloy-4 alloy by MS method, after various autoclave periods: (**a**) 0 h, (**b**) 504 h, (**c**) 1512 h, (**d**) 3024 h.

A qualitative evaluation of the hydride evolution with autoclave testing time was realized and the results are presented in Figure 3.

From Figure 3, we can notice the presence of some acicular hydrides, with a relatively uniform distribution and a very-low-density increase with the autoclave testing time.

**Figure 4:**
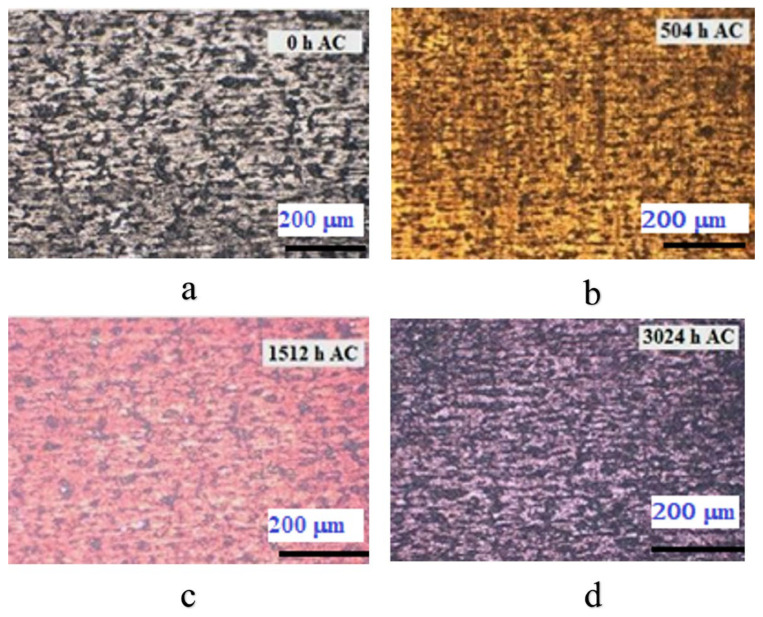
Surface morphologies of Cr-coated Zircaloy-4 alloy by MS method, after various autoclave periods: (**a**) 0 h, (**b**) 504 h, (**c**) 1512 h, (**d**) 3024 h.

Next, Figure 4 presents the morphology of Cr-coated Zircaloy-4 alloy samples according to the autoclave testing period.

As Figure 4 shows, a change in the oxide color with autoclave testing time can be observed. According to the specialized literature [27], it was noticed that the gold color indicates a thickness of less than 100 nm of the Cr_2_O_3_ layer, and the change to purple/blue is given by the increase in the thickness of the chromium oxide layer (of the order of hundreds of nanometers).

**Figure 5:**
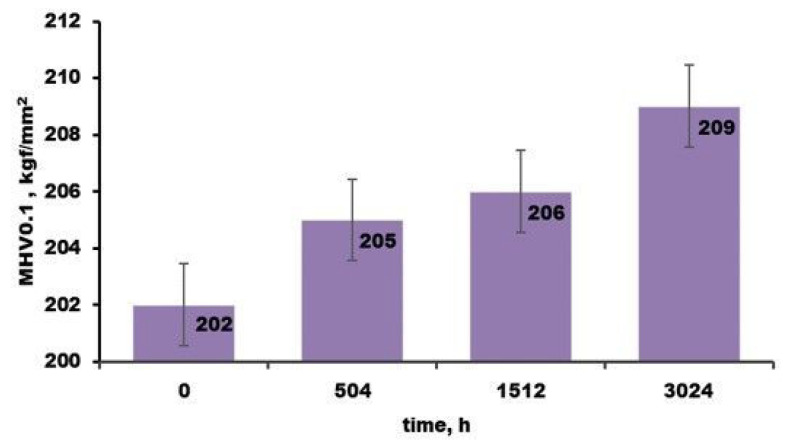
Vickers microhardness variation for autoclaved Zircaloy-4 alloy samples covered with Cr.

Vickers microhardness tests (MHV0.1) have been applied to assess the influence of the autoclave test on the microstructural properties of the coated Zircaloy-4 alloy. The test used a load of 0.1 Kgf (100 gf) [28] and it was applied in the cross-section on embedded samples in cupric resin, Figure 5.

Vickers microhardness results indicate a slight increase with the autoclave testing time [29]. However, we noticed that the values are located on a limited domain (202–209 Kgf/mm^2^), which demonstrates that there were no induced significant modifications during the autoclave testing.

#### 3.2.2. Scanning Electron Microscopy (SEM) Measurements

**Figure 6:**
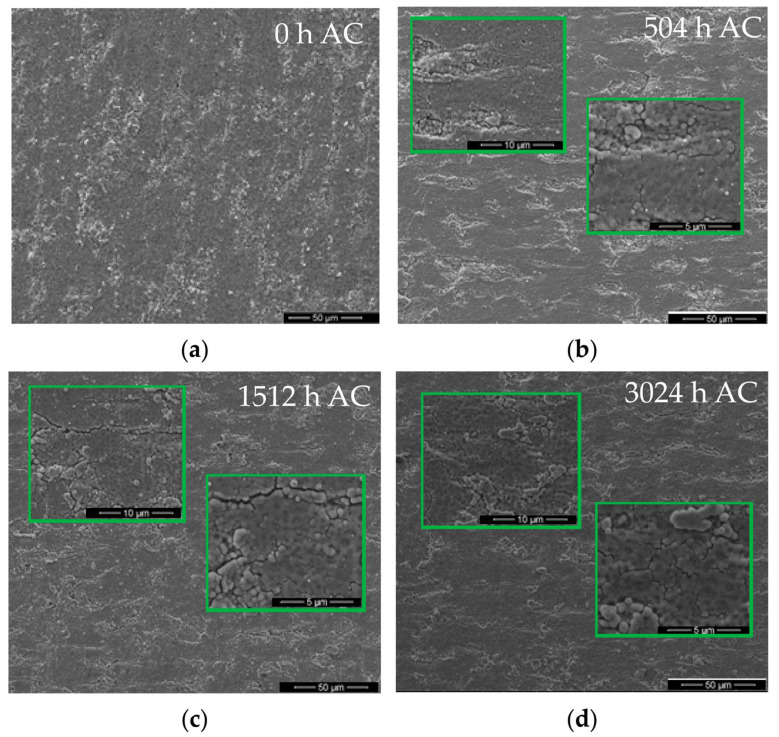
SEM micrographs illustrating surface morphology for Cr-coated Zircaloy-4 samples, after various autoclave periods: (**a**) 0 h, (**b**) 504 h, (**c**) 1512 h, (**d**) 3024 h.

Surface morphologies for the as-coated and autoclaved chromium-coated Zircaloy-4 alloy are presented in Figure 6.

From Figure 6, it is noticed that the coatings are still adherent, and they are keeping the initial morphological characteristics during the autoclave testing. There were no identified areas with spallation, cracking, or possible forms of localized corrosion.

**Figure 7:**
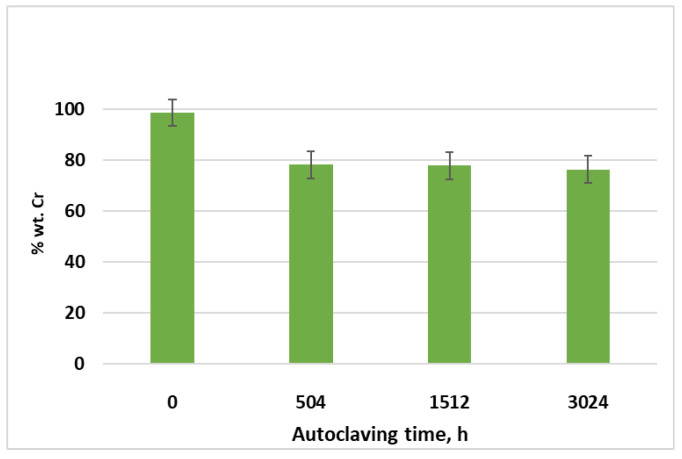
Cr concentration evolution with autoclaving time.

Cr concentration evolution on the coating surface with autoclave testing time was assessed by the EDS analysis, Figure 7.

After 504 h of autoclave testing, it was observed that the higher Cr concentration decreased, by around 20%. During the next autoclave testing period, it was noticed that the minor Cr concentration decreased, by about 1–2%. Consequently, it can be concluded that the coatings performed well in the corrosion testing environment.

**Figure 8:**
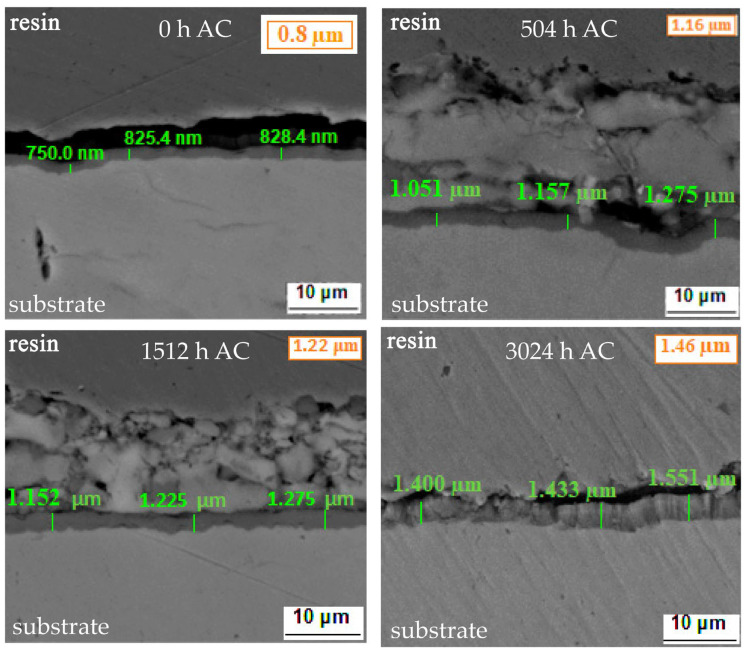
SEM cross-section micrographs for as-coated and autoclaved Cr-coated Zircaloy-4 alloy, after various autoclave periods.

In Figure 8, SEM cross-section images of the coatings are shown. The Zircaloy-4 samples that were coated with Cr using the MS procedure and autoclaved for various periods underwent integrity assessment and layer thickness evaluations.

For the as-coated sample, an adherent layer was measured, with an average thickness of approximately 0.8 µm. Measurements of the layer of the autoclaved samples show an increase in thickness, in agreement with the gravimetric measurements. It was not possible to differentiate the measured layer in metallic Cr and Cr_2_O_3_, this being a subject that is desired to be realized soon, with new generations of Cr coatings.

**Figure 9:**
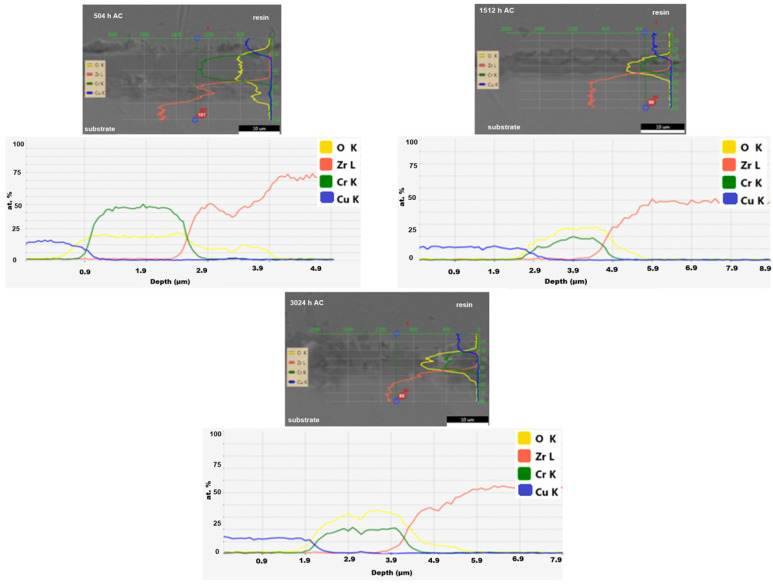
EDS line scan investigation of Cr coatings applied using MS technique on Zircaloy-4 substrate following different autoclave periods.

The variation in the elements of interest in the cross-section was investigated on the Cr-coated Zircaloy-4 samples deposited by the MS method, and the results are shown in Figure 9.

After 504 h of autoclave testing, the spectra show that in the region corresponding to chromium coating, chromium is found in a higher concentration than oxygen. It also can be seen that both Cr and O present stable behavior in this area. For the following test periods, the change in the ratio of recorded concentrations was identified, in favor of increasing the concentration of oxygen compared to that of chromium, which indicates the acceleration of the oxidation process at the coating–medium interface, under the test conditions.

#### 3.2.3. XRD Measurements

**Figure 10:**
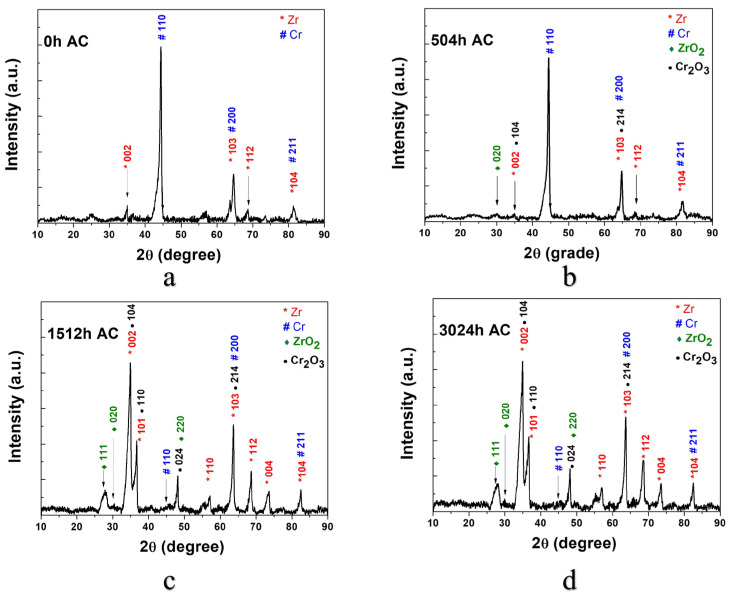
Diffraction patterns of Cr coatings deposited on Zircaloy-4 substrate by MS method, after various autoclave testing periods: 0 h (**a**), 504 h (**b**), 1512 h (**c**), and 3024 h (**d**).

The crystalline structure investigation of the coatings was carried out by the X-ray diffraction method. Figure 10 presents the diffraction patterns of the Cr-coated Zircaloy-4 alloy by the MS method.

The samples’ polycrystalline nature is emphasized by the abundance of peaks corresponding to Cr (ICDD 01-073-9565), Zr (ICDD 01-071-4633), zirconium dioxide (ICDD 01-070-2491), and chromium oxide (COD 9014850). These correspond to hexagonal zirconium P63/mmc (194), respectively, the crystalline phase of monoclinic zirconium dioxide. The latter is also identified in the case of Zircaloy-4 samples coated with Cr by the EBPVD method [30]. However, for these Zircaloy-4 samples coated by MS, an increase in the ZrO_2_ intensity peak with the autoclave testing time was not observed. It was observed that the intensity of the ZrO_2_ peak is higher after 504 h AC in the case of these samples, compared to the previously analyzed samples [30], but remains constant during the autoclave process. After 504 h of autoclave testing, the peak corresponded to pure chromium with a body-centered cubic (BCC) structure still at a high intensity. The decrease in the pure chromium peak intensity is observed after 1512 h of autoclave testing, a very weak signal corresponding to chromium being identified.

### 3.3. Evaluation of Susceptibility to Corrosion by Electrochemical Methods

The applied electrochemical methods aimed at the evaluation of the anticorrosive performance and the protective character of the Cr coatings applied on the Zircaloy-4 substrate, after testing under high temperature and pressure conditions.

#### 3.3.1. Open-Circuit Potential Tests

**Figure 11:**
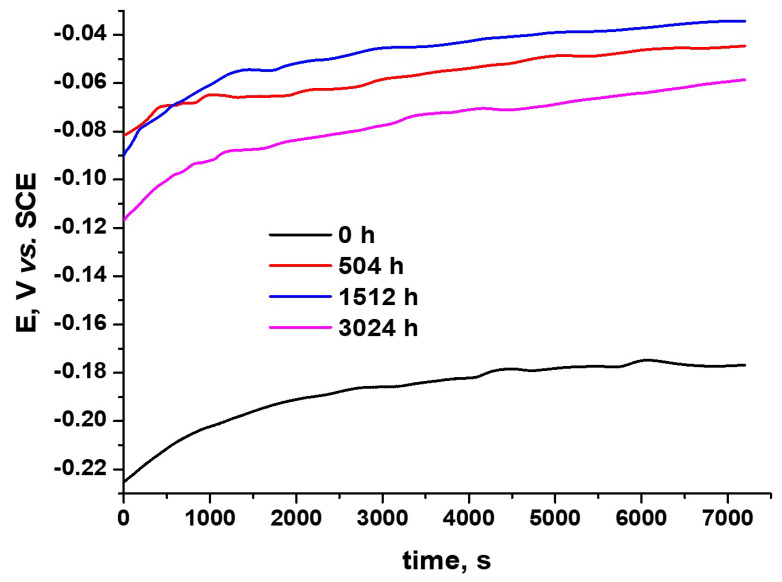
Variations in open-circuit potential Zircaloy-4 alloy coated with Cr, following several autoclave testing durations.

The generalized corrosion behavior prediction for Cr-coated Zircaloy-4 alloy samples was studied by open-circuit potential measurements. Figure 11 presents the open-circuit potential variation of Zircaloy-4 alloy samples coated with chromium using the MS method, autoclaved for different periods.

A relatively stable evolution, with an increasing potential trend, for all the tested samples was recorded. It was also noted that there were no recorded higher potential variations, which could indicate some possible discontinuities of coating. A high potential gap can be observed between the as-coated Zircaloy-4 sample and the autoclaved coated Zircaloy-4 samples, revealing the protective role of Cr_2_O_3_ [8,31,32], formed during the autoclave test. The sample autoclaved for 1512 h presented the best behavior, and a slight performance decrease is noted for the sample autoclaved for 3024 h. However, the continuous potential increase confirms that a passive oxide layer was formed and confers corrosion protection.

#### 3.3.2. Electrochemical Impedance Spectroscopy (EIS)

**Figure 12:**
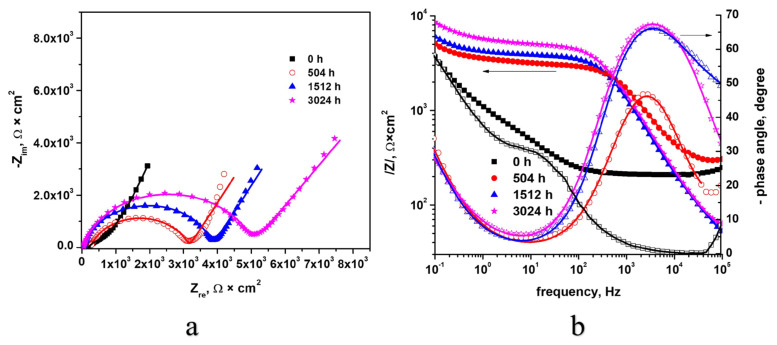
Nyquist (**a**) and Bode (**b**) diagrams for Cr-coated Zircaloy-4 alloy, after various autoclave times.

A qualitative evaluation of the protective properties of the Cr coatings was realized by EIS. The Nyquist and Bode plots (see Figure 12) present the spectra recorded at open-circuit potential, after 10 min of immersion in the test solution of Zircaloy-4 samples coated by the MS method, represented with points the experimental data and with a line the data obtained after fitting.

Based on the Nyquist diagram (Figure 12a), it is observed that there is a single capacitive semicircle, and in the case of the autoclaved samples, the formation of two capacitive semicircles was observed, following the generated interfaces. The autoclaved samples yielded bigger capacitive semicircle diameters than the as-coated sample.

According to the Nyquist diagram (Figure 12a), it can be seen in the low-frequency domain that the impedance value is higher for samples autoclaved for longer times. Taking into account the fact that the impedance at low-frequency values is related to the Faradaic process [33], we can affirm that the protective properties of the film increased with the autoclave testing time.

Based on the Bode diagrams (Figure 12b), high impedance values were determined for all autoclaved samples for distinct periods, which demonstrates a higher corrosion resistance compared to the as-coated sample.

**Figure 13:**
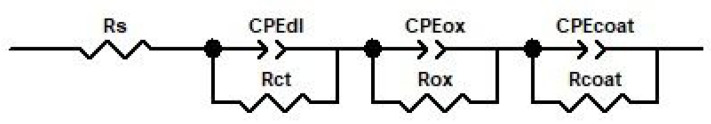
The Cr-coated Zircaloy-4 alloy’s electric equivalent circuit following different autoclave times.

The equivalent circuit model used to fit the experimental EIS data is shown in Figure 13. The following is a description of the equivalent circuit’s components: Rs stands for solution resistance between the electrode and the electrolyte; CPEox stands for the continuous phase element of the oxide layer; oxygen layer resistance is represented by Rox; coating resistance is represented by CPEcoat; coating resistance is represented by Rcoat; double-layer resistance is represented by CPEdl; and charge transfer resistance is represented by Rct.

The data summarized in Table 2 show a very good fitting of the experimental data with this equivalent circuit model. Resistance of the coating recorded the highest value for 504 h of autoclave testing; thus, for this sample, the oxides present the highest resistance to electrolyte diffusion. High values of CPE_coat_-P, CPE_ox_-P, and CPE_dl_-P are calculated, which demonstrate that both the Cr coating and the oxides are protecting the substrate [5]. From the table above, we identify the highest value of R_ct_ corresponding to the as-coated Zircaloy-4 sample. The autoclaved samples present closed R_ct_ values, the highest corresponding to the sample autoclaved for 3024 h. The values of constant phase elements are close to 1, demonstrating a capacitive character of the coating.

#### 3.3.3. Potentiodynamic Polarization Tests

**Figure 14:**
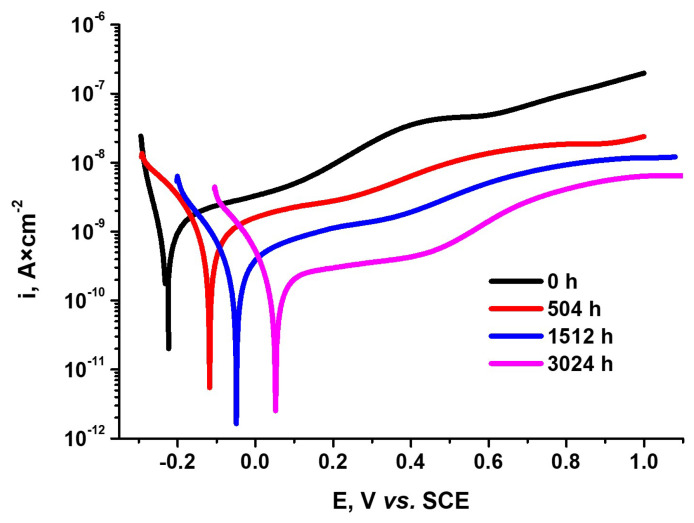
Polarization curves for Cr-coated Zircaloy-4 alloy, after various autoclave periods.

The corrosion behavior of the autoclaved Cr-coated Zircaloy-4 alloy has also been assessed by potentiodynamic polarization tests, Figure 14.

As can be seen from Figure 14, the as-coated Zy-4 sample presents more electronegative values of E_corr_ and higher corrosion currents compared to the autoclaved Cr-coated Zy-4 samples.

The electrochemical parameters specific to the corrosion process have been calculated using two methods, polarization resistance and Tafel slope extrapolation. The main parameters are corrosion potential (E_corr_), corrosion rate (V_corr_), corrosion current (i_corr_), polarization resistance (R_p_), coating porosity coefficient (P), and coating protection efficiency (P_i_) and are included in Table 3. P_i_ and P are two very important factors for coating corrosion susceptibility evaluation. As we can see from Table 3, the values of these parameters show that the coating does not lose its integrity, presenting high protection efficiency and low values of coating porosity.

The values of current densities obtained from both methods are very close, giving a high confidence in the obtained results. The current densities are in a direct ratio to corrosion rates, and both are decreasing as autoclave testing time increases. This confirms the good stability of the coating over time under autoclave test conditions.

Another important parameter that characterizes the corrosion susceptibility of the samples is the polarization resistance. We see that there were high values recorded of about an MΩ order of magnitude for R_p_. Also, the R_p_ parameter increases with autoclaving time, showing good corrosion stability of the samples over the autoclave test period.

## 4. Conclusions

The Zircaloy-4 alloy coated with a thin chromium coating was long-term-tested in a static autoclave while simulating a CANDU reactor’s primary circuit (lithiated water, 10 MPa, 310 °C, and pH = 10.5), and the results are presented in this study as a novelty.

Based on the gravimetric analysis, we noticed four times less gain mass compared to uncoated Zircaloy-4, indicating smaller corrosion rates.

Vickers microhardness results indicate a slight increase with the autoclave testing time, with values between 202 and 209 Kgf/mm^2^, which demonstrate that there were no significantly induced modifications during the autoclave testing.

Optical and SEM micrographs illustrate that the coatings are still adherent and they are keeping the initial morphological characteristics throughout the autoclave process.

Cr concentration evolution on the coating surface with autoclave testing time was assessed by the EDS analysis.

The SEM cross-section analysis has shown values of the thickness of the coating between 0.8 and 1.46 µm. The variation in the elements of interest in the cross-section was investigated by the EDS line scan analysis and the spectra showed that after 504 h of autoclave testing, a decrease in chromium is recorded compared to oxygen. This may indicate an acceleration in the oxidation process at the coating–medium interface, under the test conditions.

By the XRD analysis, the presence of Cr, Zr, and their oxides was highlighted.

A relatively stable evolution, with an increasing potential trend, for all the tested samples was recorded by the OCP method.

Based on the Nyquist diagram, larger capacitive semicircle diameters were measured for the autoclaved samples compared to the as-coated sample. High impedance values were determined for all autoclaved samples for distinct periods, which demonstrates a high corrosion resistance.

High values of P_i_ and P parameters show that the coatings do not lose integrity, presenting high protection efficiency and low values of coating porosity. The current densities and corrosion rates are decreasing as autoclave testing time increases. Also, polarization resistance, R_p_, is increasing with autoclave testing time. The electrochemical and non-electrochemical data above sustain the good stability of the MS advanced Cr coating over a long time in simulated conditions, under an autoclave. Such a conclusion is a useful technical contribution for selecting performant Cr coating on Zircaloy-4.

## Figures and Tables

**Table 1:** Kinetic parameters for Cr-coated Zircaloy-4 samples.

Kinetic Equation	*k_p_*	*n*	R^2^
y = 1.376 × *t*^0.167^	1.376	0.167	0.997

**Table 2:** The Cr-coated Zircaloy-4 alloy’s equivalent electrical circuit element values.

Autoclave Testing, h	0	504	1512	3024
R_s_, Ω × cm^2^	20.2 ± 0.2	17.3 ± 0.1	18.7 ± 0.1	19.3 ± 0.21
CPE_dl_-T, F × cm^−2^	3.7 × 10^−4^ ± 0.3	3.2 × 10^−7^ ± 0.2	2.6 × 10^−7^ ± 0.3	2.7 × 10^−7^ ± 0.1
CPE_dl_-P	0.64 ± 0.05	0.84 ± 0.08	0.89 ± 0.075	0.87 ± 0.05
R_ct_, Ω × cm^2^	1.8 × 10^14^ ± 0.5	2881 ± 0.3	3723 ± 0.2	4920 ± 0.1
CPE_ox_-T, F × cm^−2^	-	4.6 × 10^−4^ ± 0.4	4.1 × 10^−4^ ± 0.3	2.7 × 10^−4^ ± 0.6
CPE_ox_-P	-	0.71 ± 0.03	0.78 ± 0.05	0.64 ± 0.05
R_ox_, Ω × cm^2^	-	1.9 × 10^14^	7.5 × 10^18^	7.8 × 10^19^
CPE_coat_-T, F × cm^−2^	7.1 × 10^−6^ ± 0.1	7.8 × 10^−6^ ± 0.1	4.2 × 10^−5^ ± 0.2	1.1 × 10^−4^ ± 0.2
CPE_coat_-P	0.79 ± 0.03	0.99 ± 0.07	0.87 ± 0.05	0.69 ± 0.02
R_coat_, Ω × cm^2^	201.2 ± 12.4	267.1 ± 12.5	109.1 ± 11	57.6 ± 8.2
Chi-Squared	2.3 × 10^−3^	1.3 × 10^−3^	2.5 × 10^−4^	3 × 10^−4^

**Table 3:** Polarization parameters corresponding to Cr-coated Zircaloy-4 alloy, autoclaved for different periods.

Autoclaving Time, h	Tafel Slope Method			Polarization Resistance Method		Pi (%)	P (%)
	E_corr_, mV	i_corr_, nA × cm^−2^	V_corr_, nm × year^−1^	R_p_, MΩ × cm^2^	i_corr_, nA × cm^−2^		
0	−232 ± 0.4	0.797 ± 0.003	18.3 ± 0.04	31.2 ± 0.2	0.787 ± 0.004	-	-
504	−117 ± 0.3	0.515 ± 0.003	11.82 ± 0.03	55.1 ± 0.4	0.476 ± 0.003	35.38 ± 0.03	0.0166 ± 0.02
1512	−54 ± 0.5	0.216 ± 0.002	4.96 ± 0.03	210 ± 0.5	0.229 ± 0.002	72.89 ± 0.02	6.3 × 10^−4^ ± 0.01
3024	−56 ± 0.3	0.121 ± 0.002	2.77 ± 0.02	375 ± 0.6	0.122 ± 0.002	84.82 ± 0.02	0.12 × 10^−4^ ± 0.01

## Data Availability

The raw data supporting the conclusions of this article will be made available by the authors on request.

## References

[1] Rawat M., Das A., Shukla D.K., Rajput P., Chettah A., Phase D.M., Ramola R.C., Singh F. (2016). Micro-Raman and electronic structure study on kinetics of electronic excitations induced monoclinic-to-tetragonal phase transition in zirconium oxide films. RSC Adv..

[2] Wang Y.B., Zheng Y.F., Wei S.C., Li M. (2011). In vitro study on Zr-based bulk metallic glasses as potential biomaterials. J. Biomed. Mater. Res. B Appl. Biomater..

[3] Ramonti D., Gomez Sanchez A., Milosev I., Demetrescu I., Cere S. (2016). Effect of anodization on the surface characteristics and electrochemical behaviour of zirconium in artificial saliva. Mater. Sci. Eng. C.

[4] Kashkarov E., Gusey K., Kudijarov V., Kurdyumov N., Sidelev D. (2023). Hydrogenation Behavior of Cr-Coated Resistance Upset Welds of E110 Zirconium Alloy. Coatings.

[5] Chen H., Wang X., Zhang R. (2020). Application and Development Progress of Cr-Based Surface Coatings in Nuclear Fuel Element: I.Selection, Preparation, and Characteristics of Coating Materials. Coatings.

[6] Hussain D. (2020). Accident Tolerant Barriers for Fuel Rod Cladding in Nuclear Reactors. Ph.D. Thesis.

[7] Kashkarov E., Afornu B., Sidelev D., Krinitcyn M., Gouws V., Lider A. (2021). Recent advances in protective coatings for accident tolerant Zr-based fuel claddings. Coatings.

[8] Ma H.-B., Yan J., Zhao Y.-H., Liu T., Ren Q.-S., Liao Y.-H., Zuo J.-D., Liu G., Yao M.-Y. (2021). Oxidation behaviour of Cr-coated zirconium alloy cladding in high-temperature steam above 1200C. Mater. Degrad..

[9] Brachet J., Idarraga-Trujillo I., Le Flem M., Le Saux M., Vandenberghe V., Urvoy S., Rouesne E., Guilbert T., Toffolon Masclet C., Tupin M. (2019). Early studies on Cr-Coated Zircaloy-4 as enhanced accident tolerant nuclear fuel claddings for light water reactors. J. Nucl. Mater..

[10] Bischoff J., Delafoy C., Vauglin C., Barberis P., Roubeyrie C., Perche D., Duthoo D., Schuster F., Brachet J.-C., Schweitzer E.W. (2018). AREVA NP’s Enhanced Accident-Tolerant Fuel Developments: Focus on Cr-coated M5 Cladding. Nucl. Eng. Technol..

[11] Wei T., Zhang R., Yang H., Liu H., Qiu S., Wang Y., Du P., He K., Hu X., Dong C. (2019). Microstructure, corrosion resistance and oxidation behavior of Cr-coatings on Zircaloy-4 prepared by vacuum arc plasma deposition. Corros. Sci..

[12] Syrtanov M., Kashkarov E., Abdulmenova A., Gusev K., Sidelev D. (2023). High-Temperature Steam Oxidation of Accident-Tolerant Cr/Mo-Coated Zr Alloy at 1200–1400 °C. Coatings.

[13] Sevecek M., Gurgen A., Phillips B., Che Y., Wagih M., Shirvan K. (2017). Development of Cold spray Cr coated fuel cladding with enhanced accident tolerance. Nucl. Eng. Technol..

[14] Huang J., Zou S., Xiao W., Yang C., Tang D., Yu H., Zhang L., Zhang K. (2021). Influence of arc current on microstructure of Cr coating for Zr-4 alloy prepared by multi-arc ion plating via EBSD. Mater. Charact..

[15] Krejci J., Kabatova J., Manoch F., Koci J., Cvercek L., Malek J., Krum S., Sutta P., Bublikova P., Halodova P. (2020). Development and testing of multicomponent fuel cladding with enhanced accidental performance. Nucl. Eng. Technol..

[16] Yang J., Steinbruck M., Tang C., Grobe M., Liu J., Zhang J., Yun D., Wang S. (2021). Review on chromium coated zirconium alloy accident tolerant fuel cladding. J. Alloys Compd..

[17] Duarte T., Meyer Y.A., Osório W.R. (2022). The Holes of Zn Phosphate and Hot Dip Galvanizing on Electrochemical Behaviors of Multicoatings on Steel Substrates. Metals.

[18] Meyer Y.A., Menezes I., Bonatti R.S., Bortolozo A.D., Osório W.R. (2022). EIS investigation of the corrosion behavior of steel bars embedded into modified concretes with eggshell contentes. Metals.

[19] Hirschorn B., Orazem M.E., Tribollet B., Vivier V., Frateur I., Musiani M. (2010). Determination of effective capacitance and film thickness from constant-phase-element parameters. Electrochim. Acta.

[20] Hirschorn B., Orazem M.E., Tribollet B., Vivier V., Frateur I., Musiani M. (2010). Constant-Phase-Element Behavior Caused by Resistivity Distributions in Films. J. Electrochem. Soc..

[21] Diniasi D., Golgovici F., Marin A.H., Negrea A.D., Fulger M., Demetrescu I. (2021). Long-term corrosion testing of Zy-4 in a LiOH solution under high pressure and temperature conditions. Materials.

[22] Yung T., Lu W., Tsai K., Chuang C., Chen P. (2022). Oxidation behaviour characterization of Zircaloy-4 cladding with different hydrogen concentrations at 500–800C in an ambient atmosphere. Materials.

[23] Zino R., Chosson R., Ollivier M., Serris E., Favergeon L. (2021). Parallel mechanism of growth of the oxide and α-Zr(O) layers on Zircaloy-4 oxidized in steam at high temperatures. Corr. Sci..

[24] Bossis P., Lelievre G., Barberis P., Ilitis X., LeFebvre F. (2000). Multi-Scale Characterization of the Metal-Oxide Interface of Zirconium Alloys.

[25] De Oliveira L., da Silva R., dos Santos D., Ribeiro R. (2017). Hydrogen kinetics and hydride formation effect on Zr-1Nb and Zr-1Nb-1Sn-0.1Fe alloys for nuclear application. Mater. Res..

[26] Maric M., Thomas R., Nunez-Iglesias J., Atkinson M., Bertsch J., Frankel P., Race C., Barberis P., Bourlier F., Preuss M. (2022). A novel method for radial hydride analysis in zirconium alloys: HAPPy. J. Nucl. Mater..

[27] Wang Z., Garbe U., Li H., Harrison R., Kaestner A., Lehmann E. (2013). Observations on the Zirconium Hydride Precipitation and Distribution in Zircaloy-4. Metall. Mater. Trans. B.

[28] (2022). Standard Test Method for Microhardness of Materials.

[29] Ogawa Y., Takakuwa O., Tsuzaki K. (2023). Solid-solution hardening by hydrogen in Fe-Cr-Ni based austenitic steel: Temperature and strain rate effects. J. Mater. Sci..

[30] Diniasi D., Fulger M., Butoi B., Dinca P., Golgovici F. (2023). Accident tolerant barriers for fuel rod cladding of CANDU nuclear reactor. Coatings.

[31] Umretiya R., Elward B., Lee D., Anderson M., Rebak R., Rojas J. (2020). Mechanical and chemical properties of PVD and Cold Spray Cr-coatings on Zircaloy-4. J. Nucl. Mater..

[32] Hazan J., Gauthier A., Pouillier E., Shirvan K. (2021). Semi-integral LOCA test of cold-spray chromium coated zircaloy-4 accident tolerant fuel cladding. J. Nucl. Mater..

[33] OECD (2018). State-of-the-Art Report on Light Water Reactor Accident-Tolerant Fuels.

